# Humanized anti CCR4 antibody KW0761 targets HTLV-1 infected CD4+ CCR4+ and CD8+CCR4+ T cells to treat HAM/TSP

**DOI:** 10.1186/1742-4690-12-S1-O23

**Published:** 2015-08-28

**Authors:** Yoshihisa Yamano, Junji Yamauchi, Ariella Coler-Reilly, Tomoo Sato, Natsumi Araya, Naoko Yagishita, Yasuo Kunitomo, Katsunori Takahashi, Yuetsu Tanaka, Hisanao Akiyama, Yasuhiro Hasegawa, Atae Utsunomiya

**Affiliations:** 1 Department of Rare Diseases Research, Institute of Medical Science, St. Marianna University School of Medicine, Kawasaki, Kanagawa, Japan; 2 Department of Immunology, Graduate School of Medicine, University of the Ryukyus, Okinawa, Japan; 3 Division of Neurology, Department of Internal Medicine, St. Marianna University School of Medicine, Kawasaki, Kanagawa, Japan; 4 Department of Hematology, Imamura Bun-in Hospital, Kagoshima, Japan

## 

Human T-lymphotropic virus type I (HTLV-1) can cause HTLV-1-associated myelopathy/tropical spastic paraparesis (HAM/TSP) and adult T cell leukemia/lymphoma (ATL). Since the prognosis for HAM/TSP patients is extremely poor, there is a strong demand for a novel therapeutic strategy, especially one which would effectively reduce HTLV-1 proviral load, which is well-correlated with disease prognosis. CD4+CCR4+ T cells are the main HTLV-1 reservoir, and the defucosylated humanized anti-CCR4 antibody KW0761 has been approved in Japan as a treatment for ATL. KW0761 strongly binds to Fcγ receptor IIIa (FcγRIIIa) on natural killer cells and elicits powerful antibody-dependent cellular cytotoxicity (ADCC) against the CCR4+ cells. In this study, we evaluated KW0761 as a treatment for HAM/TSP using primarily ex vivo cell cultures. In addition, given that KW0761 would also target CD8+CCR4+ T cells, we sought to determine how this would likely affect HAM/TSP patients by elucidating the role of CD8+CCR4+ T cells in HAM/TSP pathogenesis. When applied to cultures of PBMCs from HAM/TSP patients (n=11), KW0761 effectively reduced HTLV-1 proviral load (56.4% mean reduction at 0.01μg/mL), spontaneous proliferation, and production of pro-inflammatory cytokines including IFN-γ. Like CD4+CCR4+ T cells, CD8+CCR4+ T cells from HAM/TSP patients exhibited high proviral loads and spontaneous IFN-γ production, unlike their CCR4-counterparts. CD8+CCR4+ T cells from HAM/TSP patients contained more IFN-γ+ cells and less IL-4+ cells than those from healthy donors. Notably, Tax-specific cytotoxic T lymphocytes that may help control the HTLV-1 infection were overwhelmingly CCR4-. In conclusion, we determined that CD8+CCR4+ T cells as well as CD4+CCR4+ T cells are prime therapeutic targets for treating HAM/TSP, and that KW0761 shows promise as a new treatment. Based on the results of this study, we have begun conducting an investigator -led Phase I/IIa clinical trial to test the safety and efficacy of KW0761 on HAM/TSP patients (UMIN000012655).

